# Rapid and visual detection of Tacheng tick virus 1 using loop-mediated isothermal amplification technique

**DOI:** 10.3389/fcimb.2025.1660327

**Published:** 2026-01-07

**Authors:** Qiqi Guo, Zheng Gui, Yuanning Ren, Ziyan Liu, Ning Liu, Liang Li, Zedong Wang, Jingfeng Yu

**Affiliations:** 1 College of Basic Medicine, Inner Mongolia Medical University, Hohhot, Inner Mongolia Autonomous Region, China; 2 Department of Infectious Diseases, Center of Infectious Diseases and Pathogen Biology, State Key Laboratory of Zoonotic Diseases, The First Hospital of Jilin University, Changchun, Jilin, China; 3 State Key Laboratory of Pathogen and Biosecurity, Changchun Veterinary Research Institute, Chinese Academy of Agricultural Sciences, Changchun, Jilin, China

**Keywords:** Tacheng tick virus 1 (TcTV-1), loop-mediated isothermal amplification (LAMP), ticks, tick-borne virus, China

## Abstract

**Introduction:**

Tacheng tick virus 1 (TcTV-1) is an emerging tick-borne nairovirus associated with human febrile illness. To date, no reliable detection method for TcTV-1 has been established. In this study, we developed and evaluated a rapid loop-mediated isothermal amplification (LAMP) assay for the detection of TcTV-1.

**Methods:**

The primers were designed based on the nucleocapsid protein (NP) gene of TcTV-1. Sensitivity was assessed using ten-fold serial dilutions of recombinant plasmids containing the target sequence. Specificity was evaluated using cDNA from Songling virus (SGLV), Yezo virus (YEZV), Tick-borne encephalitis virus (TBEV), Severe fever with thrombocytopenia syndrome virus (SFTSV), and Beiji nairovirus (BJNV). The assay was validated using field-collected tick samples.

**Results:**

The TcTV-1-specific LAMP assay detected as few as 1×10^-1^ copies/μL within 60 minutes at 65 °C and specifically amplified TcTV-1, with no cross-reactivity to SGLV, YEZV, TBEV, SFTSV, or BJNV. Positive reactions exhibited a clear color change from purple to blue, indicating a robust colorimetric response. A total of eight tick specimens (16.0%; 95% CI: 7.2–29.1) tested positive for TcTV-1 using both the established LAMP assay and SYBR Green real time quantitative polymerase chain reaction (RT-qPCR), demonstrating 100% sensitivity, specificity, positive predictive value (PPV), negative predictive value (NPV), and accuracy for the LAMP assay.

**Discussion:**

We report a TcTV-1-specific LAMP assay with high sensitivity, specificity, and cost-effectiveness, making it a practical tool for use in field-based or resource-limited settings.

## 1 Introduction

Emerging tick-borne nairoviruses have posed a growing risk to public health (Zhou et al., 2023). Tacheng tick virus 1 (TcTV-1) is a novel tick-borne virus belonging to the *Orthonairovirus* genus in the *Nairoviridae* family, and was first identified in *Dermacentor marginatus* ticks in Xinjiang, China (Li et al., 2015). Subsequently, TcTV-1 was identified as being associated with human febrile illness and rashes in the same region (Liu et al., 2020). To date, the virus has been detected in ticks, livestock, and rodents in China, as well as in ticks from Turkey and Poland (Liu et al., 2020; Dincer et al., 2023; Ergunay et al., 2023; Ji et al., 2023). These findings demonstrate the extensive geographic distribution and broad host range of TcTV-1, underscoring the importance of active surveillance in endemic regions. However, no rapid and reliable detection method for TcTV-1 has been developed.

LAMP is a nucleic acid amplification method that uses specific primers and a strand-displacing DNA polymerase to amplify target sequences rapidly under isothermal conditions, without requiring thermal cycling (Soroka et al., 2021). The LAMP method offers a simple diagnostic approach that needs merely standard equipment (water bath/heat block) to perform isothermal amplification of cDNA. The change of color (from violet to azure) can be visually observed without the need for electrophoresis, making it highly suitable for on-site analysis. These advantages, compared to conventional PCR assays, effectively meet the requirements of field testing, enabling its application in the detection of various viruses, including Dengue virus, Zika virus, Tick-borne encephalitis virus, and other emerging viruses (Hayasaka et al., 2013; Kim et al., 2018; Silva et al., 2019; Heithoff et al., 2022). In this study, we developed a TcTV-1-specific LAMP assay with high sensitivity and specificity, providing a rapid, simple, and visual method for virus detection.

## 2 Materials and methods

### 2.1 Samples and RNA extraction

Tick samples positive for TcTV-1, SGLV, YEZV, TBEV, SFTSV, and BJNV were stored in the laboratory at −80°C until use. In April 2025, a total of 50 questing *Dermacentor marginatus* ticks were collected from Xinjiang, China. RNA was extracted from homogenized tick samples and reverse-transcribed into complementary DNA (cDNA) as previously described (Gui et al., 2024).

### 2.2 Design and optimization of LAMP primers

**Figure 1:**
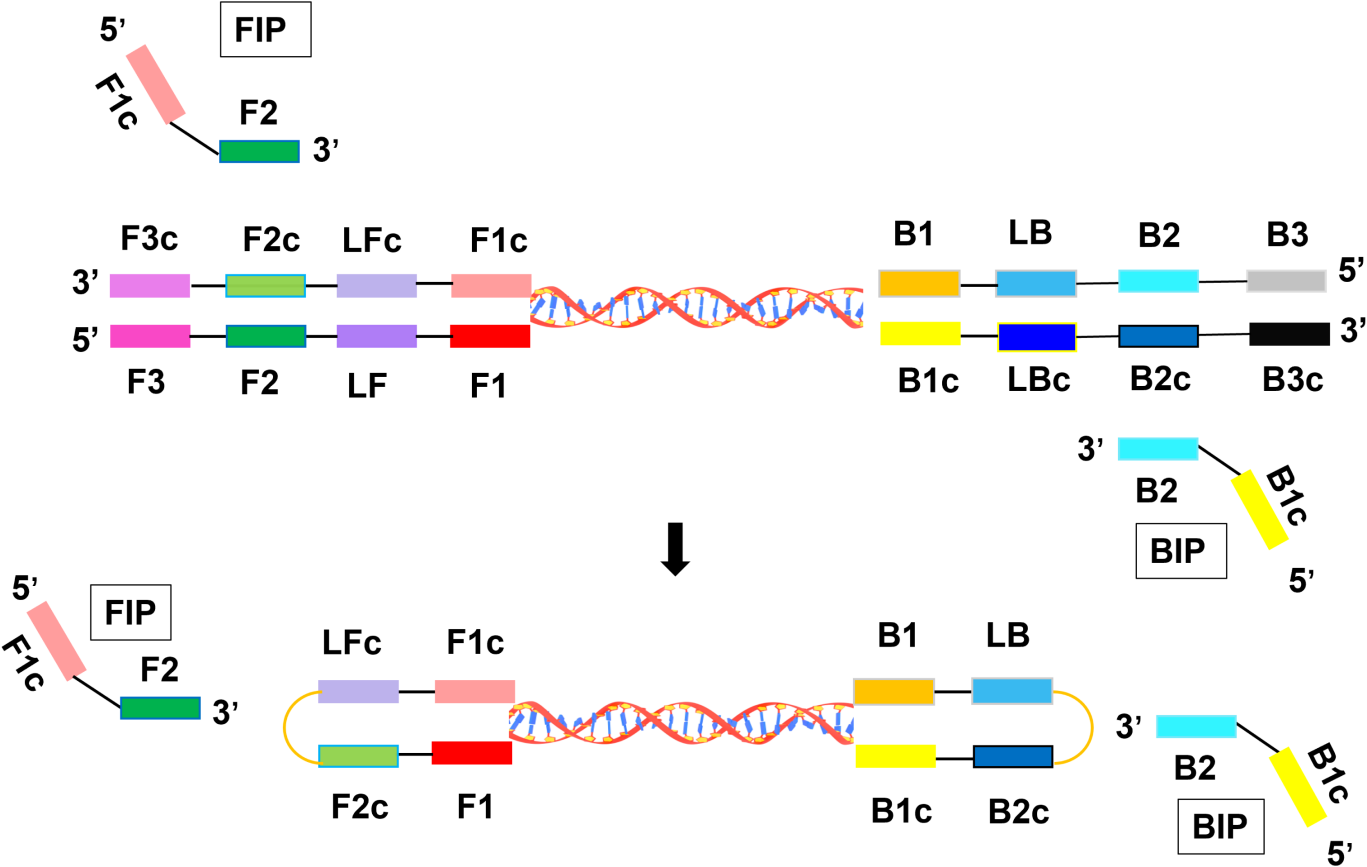
Technical principle of the LAMP assay for TcTV-1 detection.

The primers were designed based on the NP gene sequences downloaded from GenBank (**Supplementary Table S1**). To prevent cross-reactivity between TcTV-1 and the genomic sequences of other tick-borne pathogenic viruses, sequence alignment and comparison were performed using ESPript 3.0 (**Supplementary Figure S1**). The candidate primers were designed using Primer Explorer V5, with critical parameters optimized: GC content (40–60%), avoidance of non-specific binding, stability at both the 3’ and 5’ ends, melting temperature (Tm) range of 50–60°C, and absence of secondary structures such as self-dimers and cross-dimers (**Figure 1**) (Das et al., 2012; Panno et al., 2020). LAMP amplification efficiency was negatively correlated with cycle threshold; primer sets with lower thresholds were more effective. The optimal set was further confirmed in eight replicates to ensure stability.

### 2.3 Plasmid construction

The 297 bp amplification region targeted by the primers (**Supplementary Figure S2**) from the TcTV-1 JH16 strain (GenBank accession number MK554695) was commercially synthesized, digested with *Sma* I, and cloned into the pUC57 vector (Sangon Biotech, China). The recombinant plasmid was transformed into *Escherichia coli* TOP10 cells (Invitrogen), purified using a plasmid purification kit (Qiagen), and verified by Sanger sequencing. Plasmid DNA was then extracted using the TIANprep Mini Plasmid Kit (TIANGEN, China), quantified via NanoDrop 2000 spectrophotometry (ThermoFisher, USA), and copy numbers were calculated using the formula: copies/μL = [6.02 × 10²³ × concentration (g/μL)]/[average molecular weight per base pair (g/mol) × length (bp)]. The final plasmid solutions were adjusted to 1 × 10^10^ copies/μL and stored at -20°C for downstream applications.

### 2.4 LAMP reaction

The LAMP reaction system was performed as previously described (Gui et al., 2024). Briefly, the reaction mixture contained 2.5 μL of 10× isothermal amplification buffer, 1.5 μL of MgSO_4_ (100 mM), and 3.5 μL of dNTP Mix (10 mM). Amplification was initiated by adding 1 μL of Bst 2.0 WarmStart DNA polymerase (320 U/mL). Primer sets were added at final concentrations of 1.6 μM (FIP and BIP), 0.2 μM (F3 and B3), and 0.4 μM (LF and LB), with 1 μL of each primer used. The mixture was supplemented with 1 μL of EvaGreen dye, 1 μL of hydroxynaphthol blue (HNB), and 1 μL of cDNA template, and the volume was brought to 25 μL with 8.5 μL of ultrapure water. Ultrapure water was used in place of the cDNA template as a negative control.

To determine optimal conditions, amplification was performed across 63–68°C for 60 min by the Real-Time Fluorescent Quantitative PCR System (Dragonlab, China). A color change from violet to azure or a fluorescence amplification curve indicated a positive TcTV-1 result. The optimal reaction temperature is defined as the temperature at which amplification efficiency is maximized and the reaction time is minimized.

### 2.5 SYBR green RT-qPCR reaction

The SYBR green RT-qPCR was performed as the gold-standard assay, utilizing primers and reaction conditions detailed in the previous study (Liu et al., 2020). Briefly, 1 μL of each NP gene-specific primer (forward: 5′-AGAGAATGATGCTATGATTGC-3′; reverse: 5′-GAGTCCTCGTTCAACCAT-3′), 2 μL cDNA template, and 6 μL RNase-free water were added to 10 μL of 2×TB Green Premix DimerEraser (TaKaRa) to yield a final reaction volume of 20 μL. Amplification was carried out as follows: initial denaturation at 95°C for 30 s, followed by 40 cycles of 95°C for 5 s, 60°C for 30 s, and 72°C for 30 s. Viral copy numbers were calculated using the equation Y=-3.3477x+37.214 derived from the amplification plot and standard curve (**Supplementary Figure S3**).

### 2.6 Sensitivity and specificity analysis

Recombinant plasmids were serially diluted tenfold, spanning concentrations from 10^5^ to 10–^1^ copies/µL, and utilized to determine the assay’s sensitivity. Each dilution was tested in triplicate. In addition, cDNA derived from other tick-borne pathogenic viruses (SGLV, BJNV, YEZV, SFTSV, and TBEV) was tested alongside TcTV-1 to evaluate potential cross-reactivity. The LAMP reaction was performed under isothermal amplification conditions at 65°C (the optimal temperature) for 60 minutes using the Real-Time Fluorescent Quantitative PCR System (Dragonlab, China), with eight replicates. Nuclease-free ultrapure water was incorporated as a no-template control to replace cDNA in all reaction sets, enabling systematic monitoring of exogenous contamination and non-specific amplification events.

### 2.7 Detection of filed-collected ticks

The field-collected questing ticks from Xinjiang, China were tested individually using both the established TcTV-1-specific LAMP assay and the reference SYBR Green RT-qPCR method developed in a previous study (Liu et al., 2020). The diagnostic performance metrics, including prevalence, sensitivity, specificity, PPV, NPV, and accuracy, along with their 95% confidence intervals (CI), were calculated for the LAMP assay in comparison to SYBR Green RT-qPCR using an online tool, the “Diagnostic Test Evaluation Calculator” (https://www.medcalc.org/calc/diagnostic_test.php).

## 3 Results

### 3.1 Optimization of primers and reaction temperature

**Figure 2:**
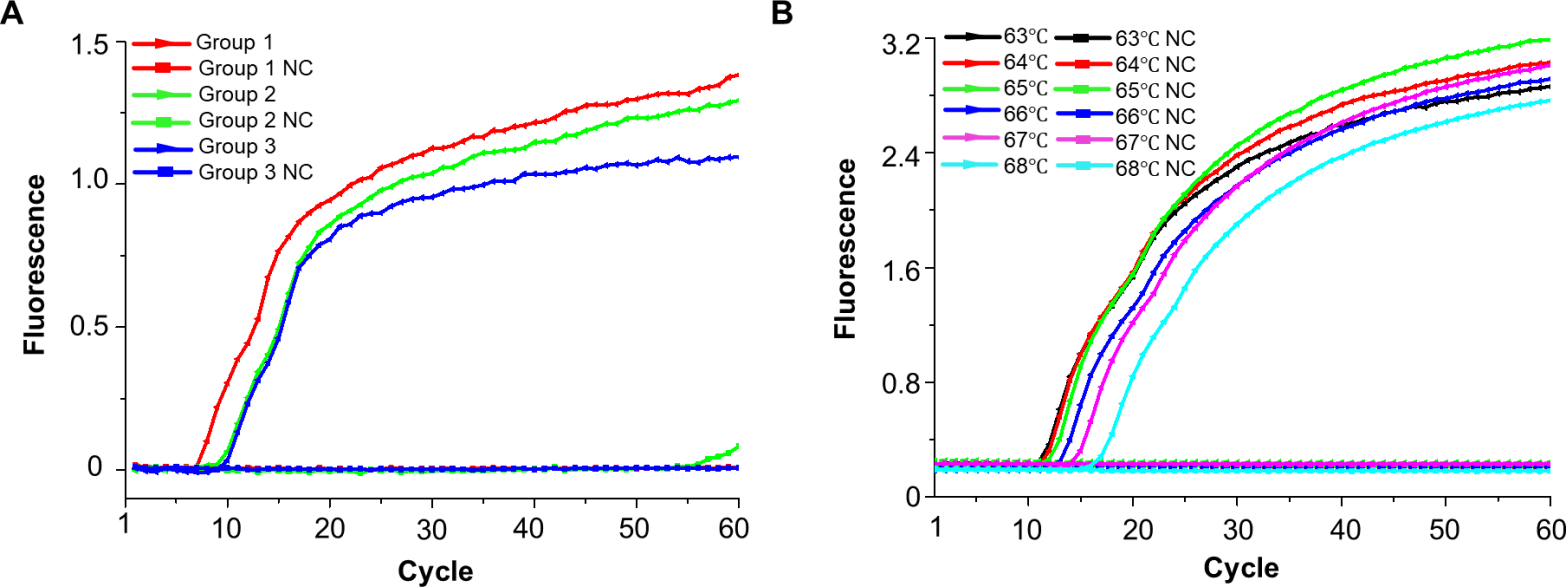
Optimization of primer sets and reaction temperature for the TcTV-1-specific LAMP assay. **(A)** Real-time fluorescence kinetics of different TcTV-1-specific LAMP primer sets. **(B)** Temperature gradient testing (63–68°C) of the LAMP assay for TcTV-1. NC, negative control.

Of the three designed LAMP primer pairs (**Supplementary Table S2**), the first set was selected for its optimal amplification kinetics and demonstrated reliable detection of the recombinant plasmid at a concentration of 1×10^6^ copies/μL (**Figure 2A**). The consistent results across eight replicate tests confirm the stability of this primer pair (**Supplementary Figure S4**). A reaction temperature of 65°C proved optimal for minimizing the detection cycle and enhancing fluorescence of TcTV-1 (**Figure 2B**, **Supplementary Figure S5**).

### 3.2 Sensitivity and specificity of TcTV-1 LAMP assay

**Figure 3:**
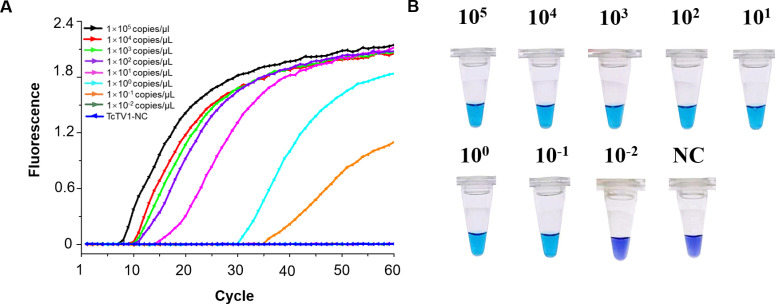
Sensitivity analysis of the TcTV-1-specific LAMP assay. **(A)** Real-time fluorescence kinetics using recombinant plasmids serially diluted from 1×10^5^ copies/μL to 1×10^-^² copies/μL. **(B)** Visualization of LAMP reaction products by gel electrophoresis. NC, negative control.

The detection limit for the LAMP assay was 1×10–^1^ copies/μL, with all positive results detected within 35 minutes (**Figure 3A**). All diluted samples exhibited a color shift from violet to azure, apart from the negative control (**Figure 3B**).

**Figure 4:**
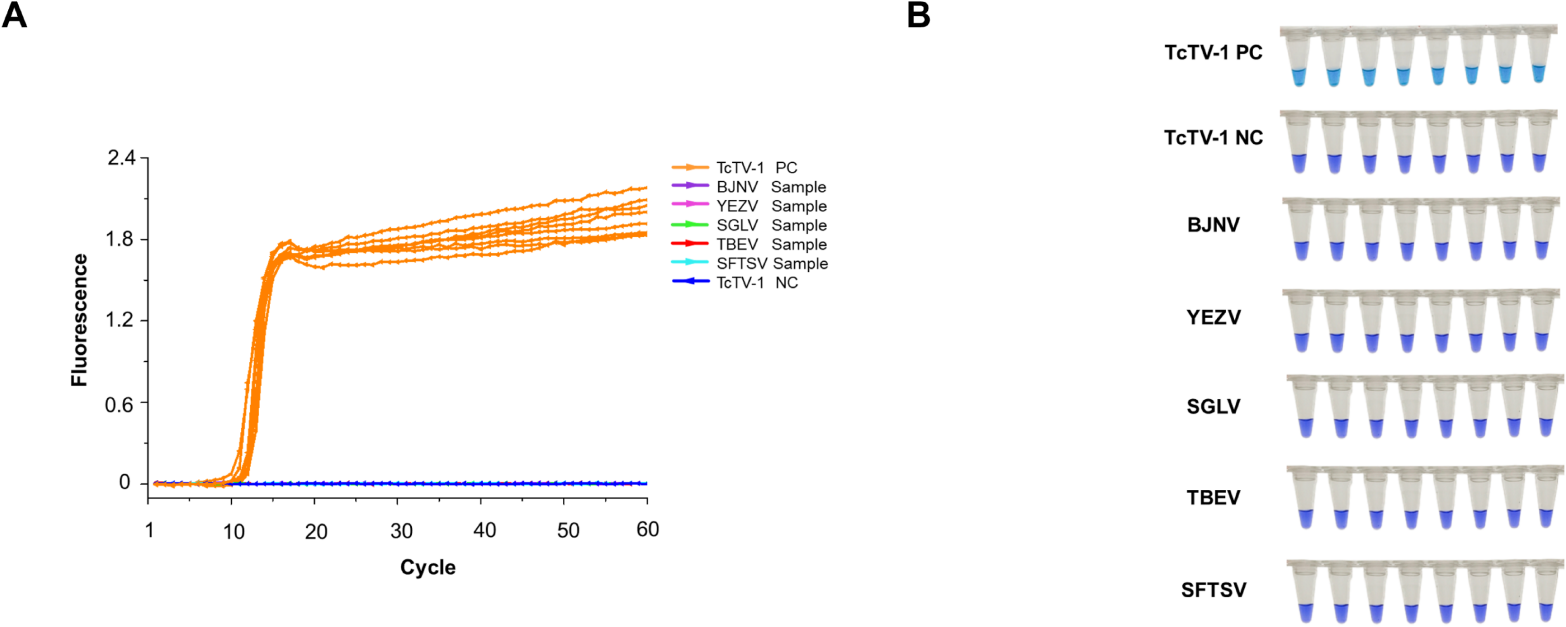
Specificity analysis of the TcTV-1-specific LAMP assay. **(A)** Real-time fluorescence kinetics for the detection of TcTV-1 and five representative tick-borne virus-positive samples. **(B)** Visualization of LAMP amplification products from TcTV-1 and five representative tick-borne virus-positive samples. BJNV, Beiji nairovirus; YEZV, Yezo virus; SGLV, Songling virus; TBEV, Tick-borne encephalitis virus; SFTSV, Severe fever with thrombocytopenia syndrome virus; PC, positive control; NC, negative control.

The LAMP assay demonstrated high specificity, detecting only the targeted TcTV-1 with no cross-reaction to other tick-borne pathogenic viruses (SGLV, BJNV, YEZV, SFTSV, and TBEV) (**Figure 4A**). Visual detection confirmed TcTV-1 via a violet-to-azure transition in its tube, whereas non-target viruses maintained purple, confirming the LAMP assay’s accurate recognition of TcTV-1 (**Figure 4B**).

### 3.3 Detection of TcTV-1 in field-collected ticks

**Figure 5:**
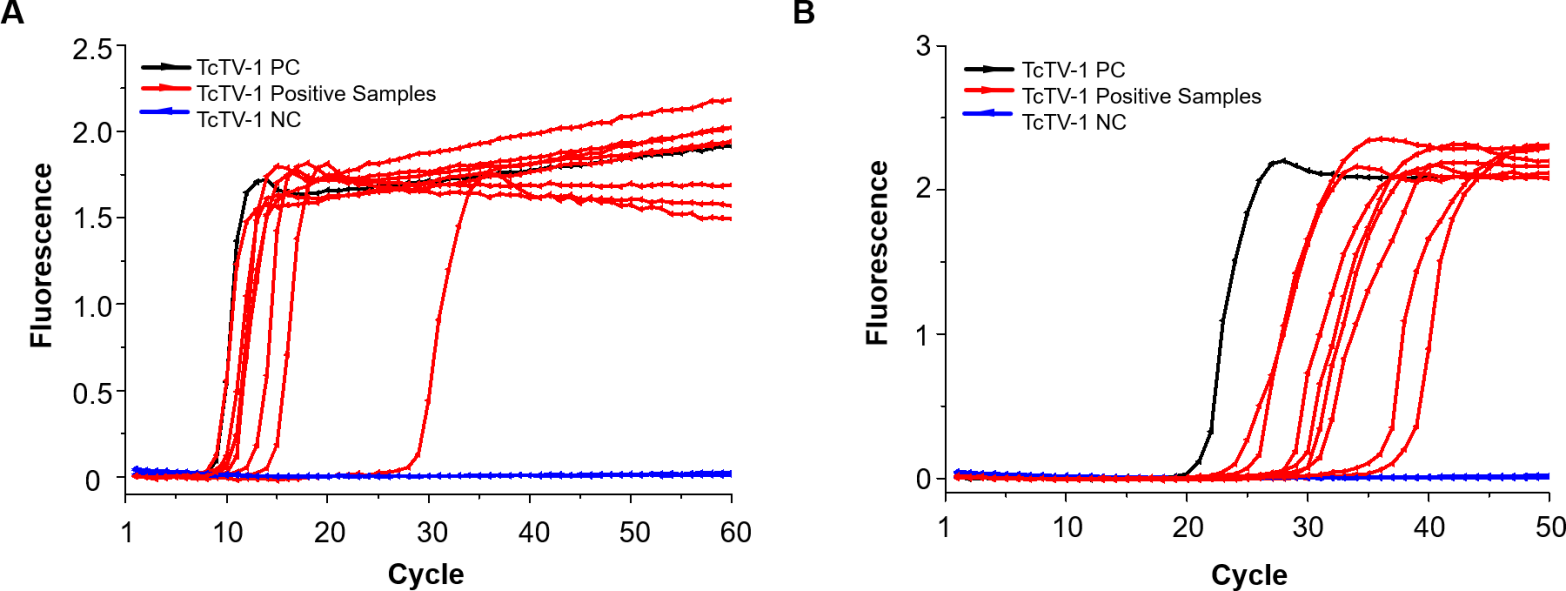
Detection of TcTV-1 in field-collected ticks from Xinjiang, China. **(A)** Detection of TcTV-1 in tick samples using the LAMP assay. **(B)** Detection of TcTV-1 in tick samples using the reference SYBR Green RT-qPCR assay. PC, positive control; NC, negative control.

A total of eight tick (16.0%; 95% CI: 7.2–29.1) individuals tested positive for TcTV-1 using the LAMP assay, with amplification times ranging from 7 to 29 (**Figure 5A**, **Supplementary Table S3**). A clear color change from violet to azure was observed in the positive samples (**Supplementary Figure S6**). Furthermore, these eight tick samples were confirmed as TcTV-1 positive by SYBR Green RT-qPCR, yielding Ct values between 24 and 36 (**Figure 5B**, **Supplementary Table S3**). The sensitivity, specificity, PPV, NPV, and accuracy of the established LAMP assay, when compared to RT-qPCR, were 100% (95% CI: 63.1–100.0), 100% (95% CI: 91.6–100.0), 100% (95% CI: 63.1–100.0), 100% (95% CI: 91.6–100.0), and 100% (95% CI: 92.9–100.0), respectively (**Supplementary Table S4**).

## 4 Discussion

TcTV-1 has been known for a decade, yet limited progress has been made in developing and validating detection methods for this virus (Liu et al., 2020; Zhang et al., 2021). Previous studies have employed various methods for detecting TcTV-1 in humans, cattle, sheep, rodents, and ticks. These methods include viral metagenomic analysis, nested PCR, SYBR Green RT-qPCR, virus isolation, indirect ELISA, and virus neutralization tests (VNT) (Liu et al., 2020; Zhang et al., 2021; Dincer et al., 2023; Ji et al., 2023). However, each of these methods has certain limitations, making it challenging to achieve rapid and visual detection. The LAMP detection method we have established addresses the shortcomings of the aforementioned methods and enables rapid visual detection of TcTV-1 nucleic acid.

In this study, we selected the SYBR Green RT-qPCR assay reported in a previous study as the reference method to evaluate the established TcTV-1-specific LAMP assay (Liu et al., 2020). The results from the detection of questing ticks demonstrated high sensitivity, specificity, and accuracy between the two assays. Although LAMP cannot quantitatively determine viral copies in the sample, it does not require specialized equipment or trained personnel and offers visual detection, making it more suitable than SYBR Green RT-qPCR for determining the presence of TcTV-1 in clinical samples.

A total of five tick-borne viruses were included to evaluated the specificity of established LAMP. Of these viruses, SGLV, YEZV, and BJNV are emerging human-pathogenic nairoviruses associated with febrile illness (Ma et al., 2021; Wang et al., 2021; Lv et al., 2023). Notably, SGLV shares a nucleotide sequence identity of over 60% with TcTV-1 (Ma et al., 2021). Additionally, as the significant tick-borne viruses, SFTSV and TBEV have also been detected in Xinjiang, China (Sun et al., 2017; Zhu et al., 2019). Our assay successfully identified TcTV-1 without cross-reactivity with other tick-borne pathogenic viruses, thereby demonstrating its high specificity for testing applications.

Our study has several limitations. First, due to the absence of TcTV-1 positive samples from humans or livestock, the established LAMP assay was not validated using clinical specimens from these hosts. Nevertheless, the method holds promise for application in both human and veterinary diagnostics. Additionally, the method established in this study relies on amplification of sample cDNA. However, if newly developed direct RNA LAMP kits, which bypass reverse transcription, are used, the detection time could be further reduced.

In conclusion, the TcTV-1-specific LAMP assay offers a rapid, highly sensitive, and visual method for detecting TcTV-1, making it ideally suited for field or resource-limited settings. This approach enables effective molecular epidemiological monitoring of TcTV-1 infection in ticks, humans, and animals.

## Data Availability

The original contributions presented in the study are included in the article/**Supplementary Material**, further inquiries can be directed to the corresponding author/s.
