# Supplementary material for: Rapid and visual detection of Tacheng tick virus 1 using loop-mediated isothermal amplification technique
Source: Front Cell Infect Microbiol. 2026 Jan 7;15:1660327. doi: 10.3389/fcimb.2025.1660327 (PMC12819602; doi:10.3389/fcimb.2025.1660327)
Supplement: Supplementary file 1 [file Table1.docx]

**Supplementary Material**

**
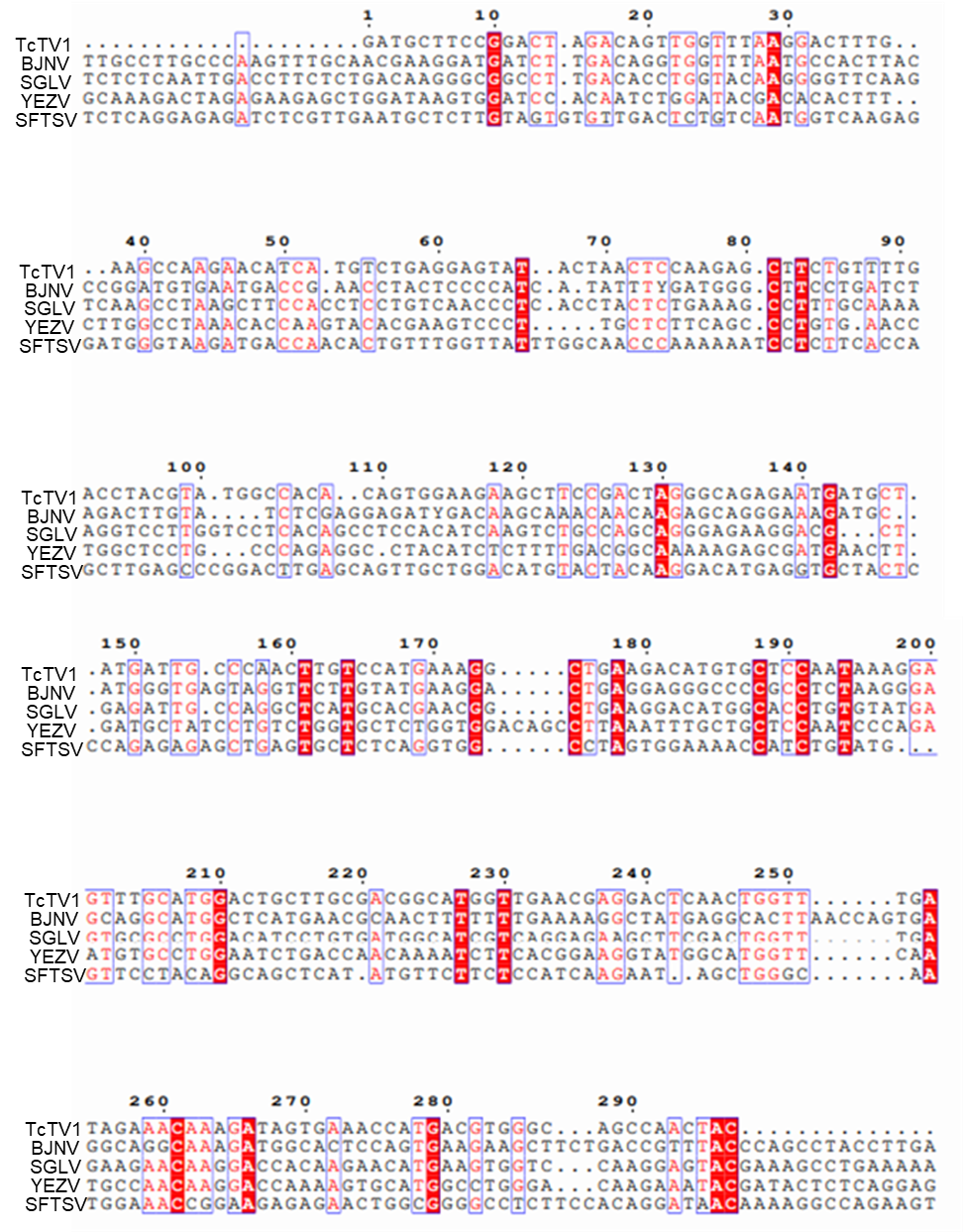
**

**Figure S1.** **Schematic diagram of sequence alignment of TcTV-1 amplification fragments with four representative viruses.** Tick-borne encephalitis virus was excluded due to the absence of the nucleocapsid protein gene. Abbreviations: BJNV, Beiji nairovirus; YEZV, Yezo virus; SGLV, Songling virus; SFTSV, Severe fever with thrombocytopenia syndrome virus.

**
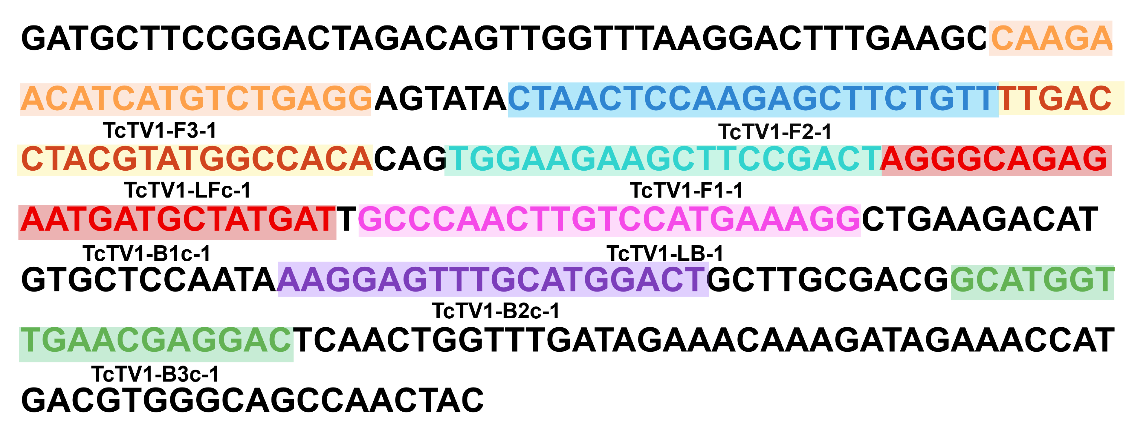
**

**Figure S2. Locations of the LAMP primer sets on the targeted fragment of the TcTV-1 nucleocapsid protein gene.**


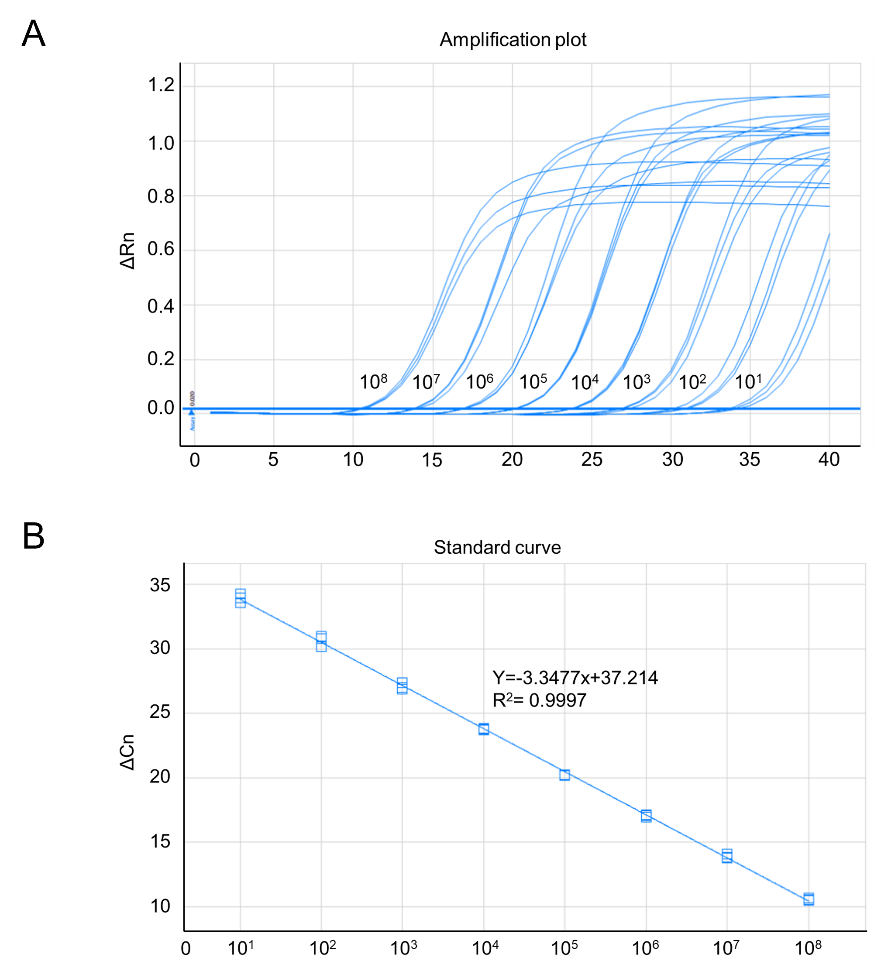


**Figure S3. Amplification plot and standard curve of the reference TcTV-1-specific SYBR Green RT-qPCR assay.**


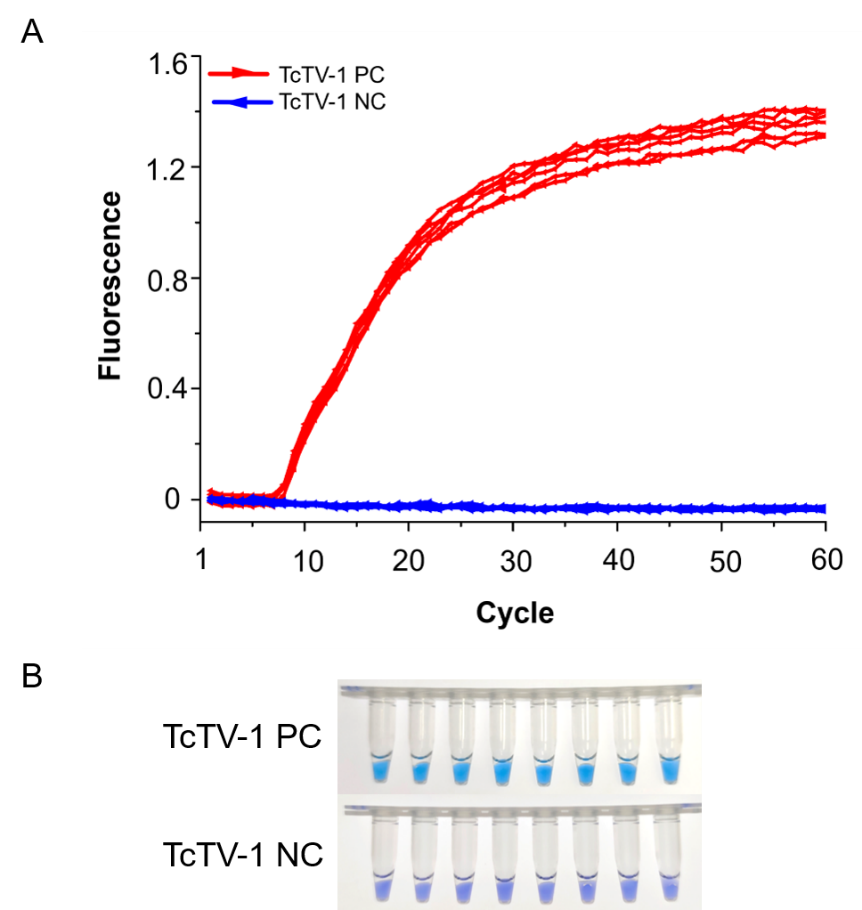


**Figure S4.** **Stability analysis of the optimal primer set.** (A) Real-time fluorescence kinetics for the detection of TcTV-1 across eight repeated experiments. (B) Visualization of LAMP amplification products from eight repeated experiments. Abbreviations: PC, positive control; NC, negative control.


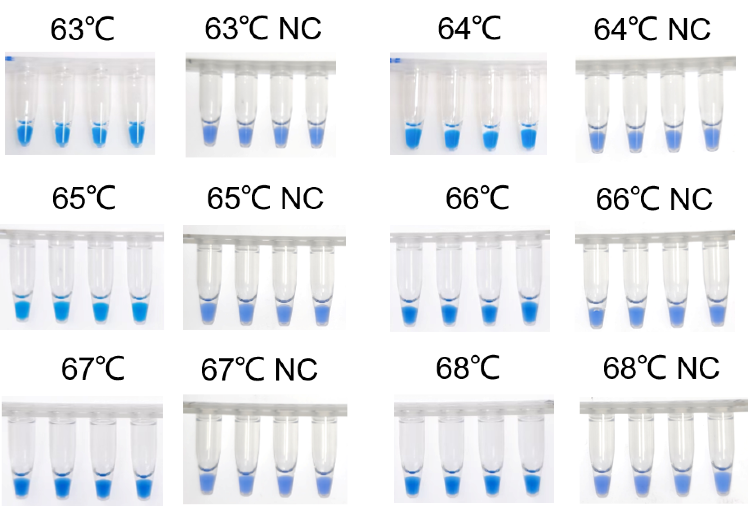


**Figure S5. Visualization results of temperature gradient testing (63–68 °C) for the TcTV-1-specific LAMP assay.** Each temperature condition was tested in triplicate. NC, negative control.


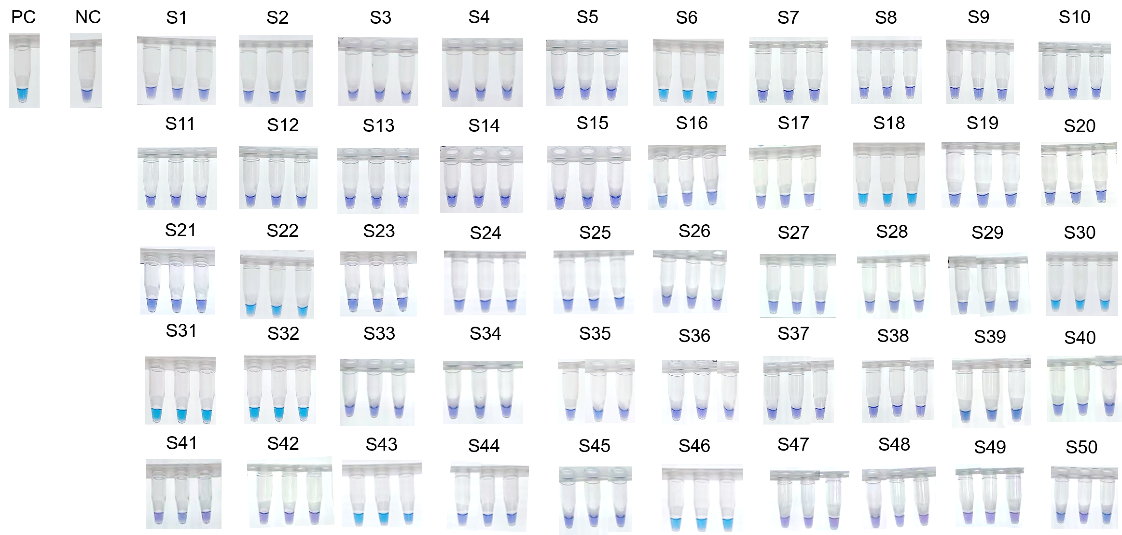


**Figure S6. Visualization results of the LAMP assay for detecting field-collected tick samples.** Each tick sample was tested in triplicate. Abbreviations: PC, positive control; NC, negative control. Sample 1-Sample 50, S1-S50.

**Table S1. Nucleocapsid protein sequences of TcTV-1 strains used for designing LAMP primer sets.**

| **ID** | **GenBank numbers** | **Strains** | **Hosts** | **Location** |
| --- | --- | --- | --- | --- |
| 1 | NC031286 | TC253 | *Dermacentor marginatus* | Tacheng, Xinjiang, China |
| 2 | MK554695 | JH16 | *Dermacentor silvarum* | Jinghe, Xinjiang, China |
| 3 | MK554694 | WQ33 | *Dermacentor nuttalli* | Wenquan, Xinjiang, China |
| 4 | MK554693 | WQ17 | *Dermacentor nuttalli* | Wenquan, Xinjiang, China |
| 5 | MK554692 | WQ9 | *Dermacentor nuttalli* | Wenquan, Xinjiang, China |
| 6 | MK554691 | WQ8 | *Dermacentor nuttalli* | Wenquan, Xinjiang, China |
| 7 | MK554690 | FH4 | *Hyalomma asiaticum* | Fuhai, Xinjiang, China |
| 8 | MK554689 | FY14 | *Dermacentor marginatus* | Fuyun, Xinjiang, China |
| 9 | MK554688 | FY7 | *Dermacentor marginatus* | Fuyun, Xinjiang, China |
| 10 | MK554687 | WS12 | *Dermacentor nuttalli* | Wusu, Xinjiang, China |
| 11 | MK554686 | SW3 | *Hyalomma asiaticum* | Shawan, Xinjiang, China |
| 12 | MK554685 | SHZ11 | Sheep | Shihezi, Xinjiang, China |
| 13 | MK554684 | SHZ7 | Sheep | Shihezi, Xinjiang, China |
| 14 | MK554683 | SHZ24 | Sheep | Shihezi, Xinjiang, China |
| 15 | MK554682 | YN2 | Cattle | Yining, Xinjiang,China |
| 16 | MK554681 | YN35 | Cattle | Yining, Xinjiang, China |
| 17 | MK554680 | QH1 | Homo sapiens | Qinghe, Xinjiang, China |
| 18 | MK639367 | 1-11 | *Dermacentor marginatus* | Almaty-Kalabak, Kazakhstan |
| 19 | MW72511 | MNS1 | Homo sapiens | Manas, Xinjiang, China |
| 20 | MK765050.1 | Bzm-1 | Homo sapiens | Qinghe, Xinjiang, China |

**Table S2. TcTV-1-specific LAMP primer sets designed in this study.**

| **Primer No.** | **Primer name** | **Sequence (5**'→**3')** |
| --- | --- | --- |
| 1 | TcTV1-FIP-1 | AGTCGGAAGCTTCTTCCACTAACTCCAAGAGCTTCTGTT |
|  | TcTV1-BIP-1 | AGGGCAGAGAATGATGCTATGATTAGTCCATGCAAACTCCTT |
|  | TcTV1-F3-1 | CAAGAACATCATGTCTGAGG |
|  | TcTV1-B3-1 | GTCCTCGTTCAACCATGC |
|  | TcTV1-LF-1  TcTV1-LB-1 | TGTGGCCATACGTAGGTCAA  GCCCAACTTGTCCATGAAAGG |
| 2 | TcTV1-FIP-2 | AATCATAGCATCATTCTCTGCCCGACCTACGTATGGCCACA |
|  | TcTV1-BIP-2 | TTGTCCATGAAAGGCTGAAGACATTGAGTCCTCGTTCAACCA |
|  | TcTV1-F3-2 | AACTCCAAGAGCTTCTGTT |
|  | TcTV1-B3-2 | TCTTTGTTTCTATCAAACCAGT |
|  | TcTV1-LF-2 | GTCGGAAGCTTCTTCCACTG |
|  | TcTV1-LB-2 | GTGCTCCAATAAAGGAGTTTGCA |
| 3 | TcTV1-FIP-3 | GGCAATCATAGCATCATTCTCTGCCCTACGTATGGCCACACA |
|  | TcTV1-BIP-3 | TTGTCCATGAAAGGCTGAAGACATTGAGTCCTCGTTCAACCA |
|  | TcTV1-F3-3 | AACTCCAAGAGCTTCTGTT |
|  | TcTV1-B3-3 | TCTTTGTTTCTATCAAACCAGT |
|  | TcTV1-LF-3 | CCTAGTCGGAAGCTTCTTCCAC |
|  | TcTV1-LB-3 | GTGCTCCAATAAAGGAGTTTGCAT |

**Table S3. Comparative analysis of the detection results from field-collected tick samples using the established TcTV-1-specific LAMP and reference SYBR Green RT-qPCR assays.**

| **Samples** | **LAMP (Ct values)** | **RT-qPCR (Ct values)** | |
| --- | --- | --- | --- |
| 1 | N/A | | N/A |
| 2 | N/A | | N/A |
| 3 | N/A | | N/A |
| 4 | N/A | | N/A |
| 5 | N/A | | N/A |
| 6 | 10.33 | | 25.60 |
| 7 | N/A | | N/A |
| 8 | N/A | | N/A |
| 9 | N/A | | N/A |
| 10 | N/A | | N/A |
| 11 | N/A | | N/A |
| 12 | N/A | | N/A |
| 13 | N/A | | N/A |
| 14 | N/A | | N/A |
| 15 | N/A | | N/A |
| 16 | N/A | | N/A |
| 17 | N/A | | N/A |
| 18 | 9.78 | | 24.29 |
| 19 | N/A | | N/A |
| 20 | N/A | | N/A |
| 21 | N/A | | N/A |
| 22 | 28.03 | | 35.31 |
| 23 | N/A | | N/A |
| 24 | N/A | | N/A |
| 25 | N/A | | N/A |
| 26 | N/A | | N/A |
| 27 | N/A | | N/A |
| 28 | N/A | | N/A |
| 29 | N/A | | N/A |
| 30 | 14.78 | | 32.91 |
| 31 | 10.57 | | 28.42 |
| 32 | N/A | | N/A |
| 33 | 12.58 | | 29.57 |
| 34 | N/A | | N/A |
| 35 | N/A | | N/A |
| 36 | N/A | | N/A |
| 37 | N/A | | N/A |
| 38 | N/A | | N/A |
| 39 | N/A | | N/A |
| 40 | N/A | | N/A |
| 41 | N/A | | N/A |
| 42 | N/A | | N/A |
| 43 | 10.97 | | 27.95 |
| 44 | N/A | | N/A |
| 45 | N/A | | N/A |
| 46 | 11.25 | | 28.33 |
| 47 | N/A | | N/A |
| 48 | N/A | | N/A |
| 49 | N/A | | N/A |
| 50 | N/A | | N/A |

**Table S4. Diagnostic performance metrics of the established TcTV-1-specific LAMP assay compared to the reference SYBR Green RT-qPCR assay for detecting field-collected tick samples.**

| **Samples** | | **Sensitivity (95% CI)** | **Specificity (95% CI)** | **Accuracy (95% CI)** | **PPV (95% CI)** | **NPV (95% CI)** |
| --- | --- | --- | --- | --- | --- | --- |
| Positive | Negative |  |  |  |  |  |
| 8 | 42 | 1.0 (63.1–100.0) | 1.0 (91.6–100.0) | 1.0 (92.9–100.0) | 1.0 (63.1–100.0) | 1.0 (91.6–100.0) |

Abbreviations: CI, confidence interval; PPV, positive predictive value; NPV, negative predictive value.
